# Supplementary figures and images for: A Framework to Explore the Knowledge Structure of Multidisciplinary Research Fields
Source: PLoS One. 2015 Apr 27;10(4):e0123537. doi: 10.1371/journal.pone.0123537 (PMC4410998; doi:10.1371/journal.pone.0123537)

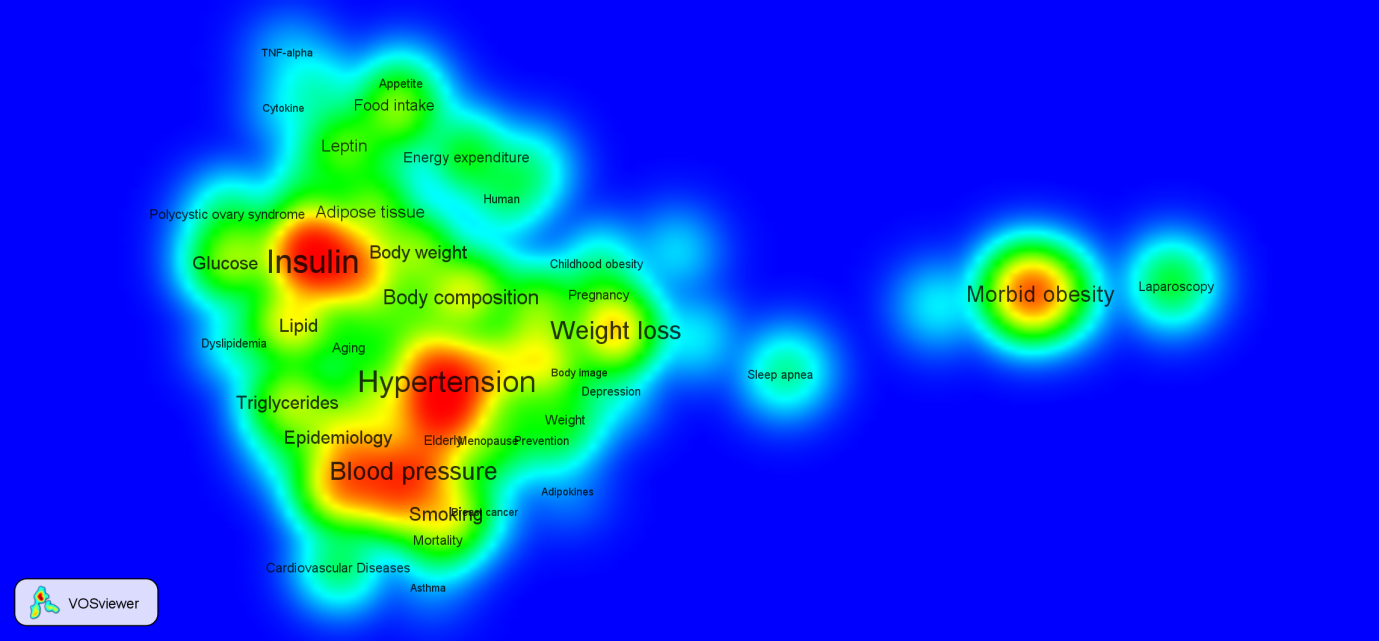

Supplement: S1 Fig — (PNG) [file pone.0123537.s001.png]

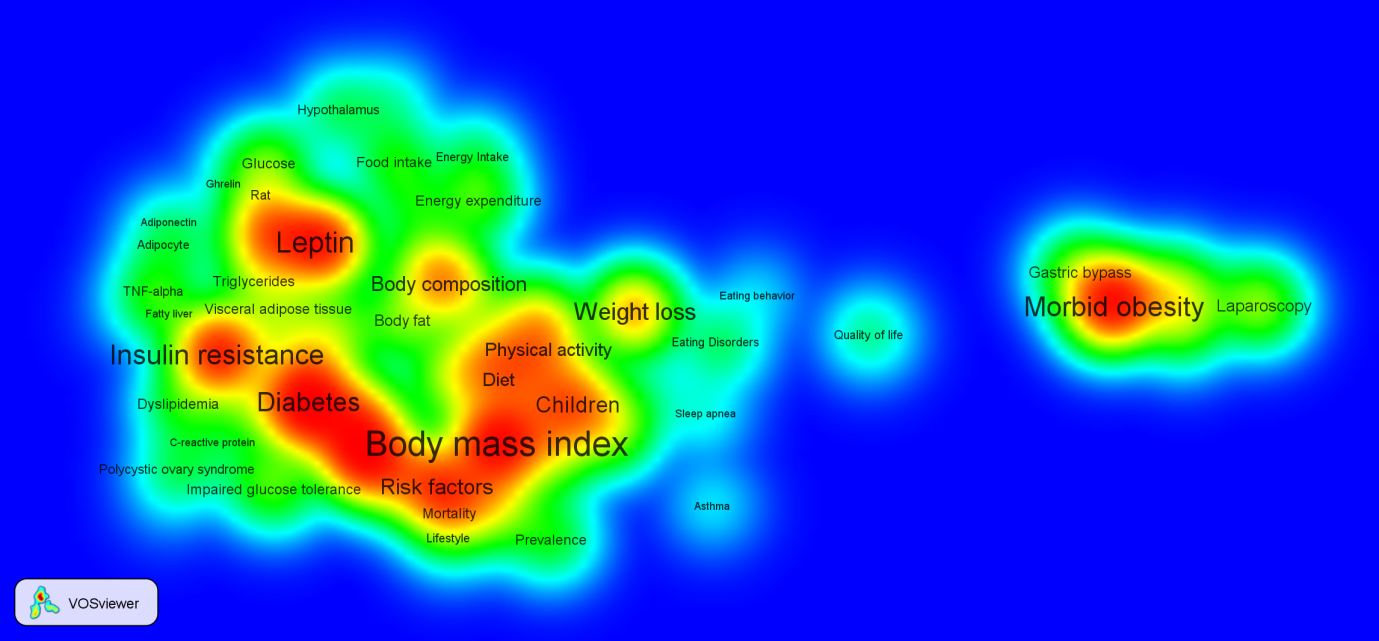

Supplement: S2 Fig — (PNG) [file pone.0123537.s002.png]

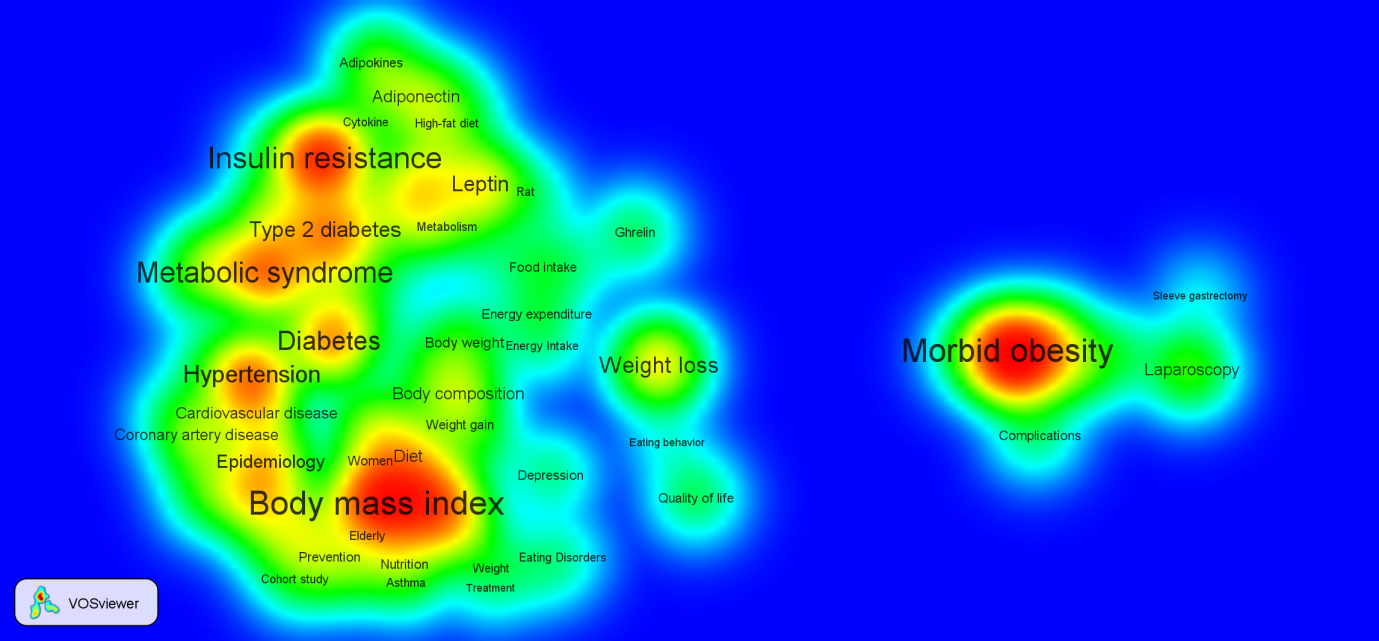

Supplement: S3 Fig — (PNG) [file pone.0123537.s003.png]

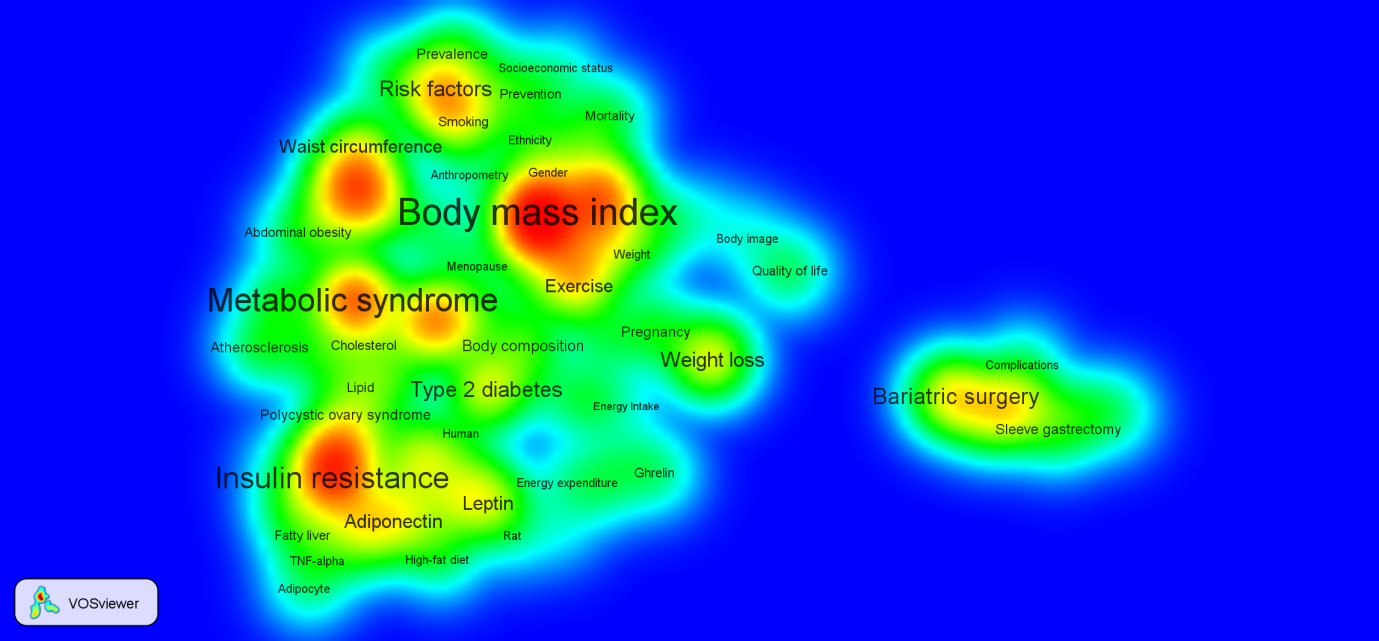

Supplement: S4 Fig — (PNG) [file pone.0123537.s004.png]
