# Supplementary material for: A Framework to Explore the Knowledge Structure of Multidisciplinary Research Fields
Source: PLoS One. 2015 Apr 27;10(4):e0123537. doi: 10.1371/journal.pone.0123537 (PMC4410998; doi:10.1371/journal.pone.0123537)
Supplement: S1 Table — Keywords with grey backgrounds appeared in all four time periods (could be in different numerical order). (DOCX) [file pone.0123537.s005.docx]

**S1 Table:** Top-50 frequently appeared keywords in the research dataset. Keywords with grey backgrounds appeared in all four time periods (could be in different numerical order).

| **ID** | **20 years** | **1993-1997** | **1998-2002** | **2003-2007** | **2008-2012** |
| --- | --- | --- | --- | --- | --- |
| 1 | Body Mass Index | Insulin | Body Mass Index | Body Mass Index | Body Mass Index |
| 2 | Insulin Resistance | Insulin Resistance | Leptin | Insulin Resistance | Metabolic Syndrome |
| 3 | Diabetes | Weight Loss | Insulin Resistance | Morbid Obesity | Diabetes |
| 4 | Metabolic Syndrome | Hypertension | Diabetes | Diabetes | Insulin Resistance |
| 5 | Children | Body Mass Index | Hypertension | Metabolic Syndrome | Children |
| 6 | Hypertension | Diabetes | Weight Loss | Bariatric Surgery | Adolescents |
| 7 | Type 2 Diabetes | Type 2 Diabetes | Morbid Obesity | Children | Type 2 Diabetes |
| 8 | Morbid Obesity | Body Composition | Insulin | Weight Loss | Bariatric Surgery |
| 9 | Weight Loss | Morbid Obesity | Type 2 Diabetes | Hypertension | Hypertension |
| 10 | Leptin | Risk Factors | Children | Leptin | Weight Loss |
| 11 | Bariatric Surgery | Blood Pressure | Risk Factors | Type 2 Diabetes | Physical Activity |
| 12 | Adolescents | Adipose Tissue | Body Composition | Risk Factors | Risk Factors |
| 13 | Risk Factors | Leptin | Adipose Tissue | Adolescents | Inflammation |
| 14 | Physical Activity | Exercise | Exercise | Physical Activity | Adipose Tissue |
| 15 | Adipose Tissue | Children | Bariatric Surgery | Body Composition | Leptin |
| 16 | Insulin | Diet | Blood Pressure | Adipose Tissue | Morbid Obesity |
| 17 | Body Composition | Body Weight | Diet | Gastric Bypass | Epidemiology |
| 18 | Epidemiology | Cholesterol | Physical Activity | Insulin | Exercise |
| 19 | Exercise | Vertical Banded Gastroplasty | Coronary Artery Disease | Epidemiology | Adiponectin |
| 20 | Inflammation | Zucker Rat | Epidemiology | Exercise | Body Composition |
| 21 | Diet | Epidemiology | Adolescents | Diet | Diet |
| 22 | Blood Pressure | Food Intake | Body Weight | Adiponectin | Childhood Obesity |
| 23 | Gastric Bypass | Coronary Artery Disease | Food Intake | Inflammation | Waist Circumference |
| 24 | Adiponectin | Smoking | Laparoscopy | Coronary Artery Disease | Cardiovascular Disease |
| 25 | Cardiovascular Disease | Triglycerides | Energy Expenditure | Blood Pressure | Gastric Bypass |
| 26 | Coronary Artery Disease | Glucose | Lipid | Body Weight | Blood Pressure |
| 27 | Body Weight | Hyperinsulinemia | Smoking | Polycystic Ovary Syndrome | Adipokines |
| 28 | Waist Circumference | Energy Expenditure | Body Fat | Cardiovascular Disease | Insulin |
| 29 | Childhood Obesity | Impaired Glucose Tolerance | Cardiovascular Disease | Laparoscopy | Pregnancy |
| 30 | Polycystic Ovary Syndrome | Fat Distribution | Hypothalamus | Prevalence | Nutrition |
| 31 | Prevalence | Lipoproteins | Women | Atherosclerosis | Prevalence |
| 32 | Nutrition | Rat | Triglycerides | Waist Circumference | Polycystic Ovary Syndrome |
| 33 | Pregnancy | Physical Activity | Cholesterol | Women | Non-Alcoholic Fatty Liver Disease |
| 34 | Atherosclerosis | Lipid | Visceral Adipose Tissue | Food Intake | Body Weight |
| 35 | Food Intake | Adolescents | Gastric Bypass | C-Reactive Protein | Coronary Artery Disease |
| 36 | Smoking | Body Fat Distribution | Metabolic Syndrome | Weight Gain | Atherosclerosis |
| 37 | Adipokines | Polycystic Ovary Syndrome | Weight Gain | Nutrition | Quality Of Life |
| 38 | Visceral Adipose Tissue | Visceral Adipose Tissue | Vertical Banded Gastroplasty | Quality Of Life | Visceral Adipose Tissue |
| 39 | Women | Insulin Sensitivity | Glucose | Smoking | Oxidative Stress |
| 40 | Weight Gain | Thermogenesis | Prevalence | Insulin Sensitivity | Depression |
| 41 | Quality Of Life | Anthropometry | Neuropeptide Y | Childhood Obesity | Prevention |
| 42 | Triglycerides | Gastric Bypass | Polycystic Ovary Syndrome | Lipid | Mortality |
| 43 | Laparoscopy | Women | Hyperinsulinemia | Triglycerides | Smoking |
| 44 | Lipid | Brown Adipose Tissue | Impaired Glucose Tolerance | Impaired Glucose Tolerance | Sleeve Gastrectomy |
| 45 | Insulin Sensitivity | Prevalence | Anthropometry | Adipocyte | Weight Gain |
| 46 | Mortality | Age | Genetics | Pregnancy | Fatty Liver |
| 47 | Cholesterol | Pregnancy | Mortality | Ghrelin | Food Intake |
| 48 | Adipocyte | Body Fat | Gastric Banding | Gastric Banding | Dyslipidemia |
| 49 | Prevention | Insulin Secretion | TNF-Alpha | Dyslipidemia | C-Reactive Protein |
| 50 | Non-Alcoholic Fatty Liver Disease | Atherosclerosis | Aging | Prevention | Women |
