# Supplementary material for: A Framework to Explore the Knowledge Structure of Multidisciplinary Research Fields
Source: PLoS One. 2015 Apr 27;10(4):e0123537. doi: 10.1371/journal.pone.0123537 (PMC4410998; doi:10.1371/journal.pone.0123537)
Supplement: S2 Table — (DOCX) [file pone.0123537.s006.docx]

**S2 Table:** Major keywords under each category.

| Diagnostic Techniques and Procedures | Body Mass Index, Blood Pressure, Body Weight, Waist Circumference, Laparoscopy, Abdominal Obesity, Anthropometry, Weight, Central Obesity, Magnetic Resonance Imaging, Glucose Tolerance Test, Body Fat Distribution, Dual-Energy X-Ray Absorptiometry, Screening |
| --- | --- |
| Endocrine System Diseases | Cushing's Syndrome, Diabetes, Type 2 diabetes, Gestational Diabetes, Diabetic Nephropathy, Prediabetes, Polycystic Ovary Syndrome, Hyperandrogenism, Ovary Cancer, Hypogonadism, Anovulation |
| Public Health | Body Mass Index, Risk Factors, Prevalence, Mortality, Anthropometry, Cohort Study, Public Health, Screening, Incidence, Comorbidity, Risk, Morbidity, Longitudinal Study, Case-Control Study |
| Peptides | Adipokines, Adiponectin, AMP-Activated Protein Kinase, AMPK, Angiotensin II, Cholecystokinin, C-Peptide, Endothelin-1, Ghrelin, Glucagon, Growth Hormone, IGF-1, IGF-I, IL-6, Insulin, Interleukin-6, Leptin |
| Metabolic Diseases | Insulin Resistance, Diabetes, Metabolic syndrome, Type 2 diabetes, Insulin Sensitivity, Dyslipidemia, Hyperinsulinemia, Hyperlipidemia, Gestational Diabetes, Hyperglycemia, Hypercholesterolemia, Hypertriglyceridemia, Lipodystrophy, Gout, Prediabetes, Hypoglycemia, Hyperinsulinism |
| Hormones | Leptin, Insulin, Adiponectin, Adipokines, Ghrelin, Glucagon-Like Peptide-1, Resistin, Testosterone, Growth Hormone, Cortisol, Hormones, Peptide YY, Glucocorticoids, Cholecystokinin, Estradiol, C-Peptide, Glucagon |
| Proteins | Adipokines, Adiponectin, AMP-Activated Protein Kinase, AMPK, Angiotensin II, Apolipoprotein A-I, Apolipoproteins, Apolipoproteins B, Apolipoproteins E, Cholecystokinin, C-Reactive Protein, Endothelin-1, Fibrinogen, High Density Lipoprotein Cholesterol, IGF-1, Insulin Receptor, Interleukin-6, Leptin, Leptin Receptor, Lipoproteins, Low Density Lipoprotein Cholesterol, Neuropeptide Y, Neuropeptides, PAI-1, POMC, PPAR, Proinsulin, Proopiomelanocortin, Resistin |
| Investigative Epidemiologic Methods | Body Weight, Waist Circumference, Anthropometry, Weight, Waist-Hip Ratio, Skinfold Thickness, Body Size, Body Height, Indirect Calorimetry, Calorimetry, Dual-Energy X-Ray Absorptiometry, Microdialysis, Dialysis, Calorimetry, Glucose Clamp Technique, Glucose Tolerance Test, Immunohistochemistry, Glucose Clamp Technique, Prevalence, Mortality, Cohort Study, Screening, Incidence, Risk, Morbidity |
| Body Constitution | Body Mass Index, Morbid Obesity, Body Composition, Body Weight, Waist Circumference, Abdominal Obesity, Weight, Central Obesity, Body Fat Distribution, Underweight, Visceral Obesity, Severe Obesity, Waist-Hip Ratio, Skinfold Thickness, Body Size, Body Height, Thinness |
| Signs and Symptoms | Inflammation, Chronic Disease, Atrial Fibrillation, Fibrosis, Postoperative Complications, Recurrence, Cirrhosis, Hyperuricemia, Chronic Diseases, Left Ventricular Hypertrophy, Gallstones, Hypertrophy, Sarcopenia, Hernia, Morbid Obesity, Weight Loss, Weight Gain, Weight, Binge Eating, Underweight, Hyperphagia, Severe Obesity, Pain, Hypoxia |
| Quality of Health Care | Risk Factors, Cohort Study, Screening, Comorbidity, Risk, Longitudinal Study, Case-Control Study, Longitudinal Studies, Prospective Study, Survey, Cross-Sectional Studies, Cross-Sectional Study, Prospective Studies, NHANES, Case-Control Studies, Comorbidities, Factor Analysis, Questionnaire, Risk Assessment |
| Heart Diseases | Coronary Artery Disease, Myocardial Infarction, Heart Failure, Left Ventricular Hypertrophy, Atrial Fibrillation, Heart Disease, Ischemic Heart Disease, Cardiomyopathy, Acute Coronary Syndrome |
| Lipids | Dietary Fat, Dietary Fats, Free Fatty Acids, Endocannabinoids, Arachidonic Acid, Triglycerides, Lipoproteins, High Density Lipoprotein Cholesterol, Apolipoproteins, Apolipoproteins B, Low Density Lipoprotein Cholesterol, Apolipoproteins E, Apolipoprotein A-I, Cholesterol, High Density Lipoprotein Cholesterol, Low Density Lipoprotein Cholesterol, Calcifediol, Cholesterol |
| Physiological Processes | Weight Loss, Body Weight, Food Intake, Weight Gain, Aging, Oxidative Stress, Weight, Growth, Thermogenesis, Breastfeeding, Eating, Body Size, Body Height, Fetal Programming, Longevity, Appetite Regulation |
| Behavior | Physical activity, Smoking, Depression, Eating Behavior, Health Behavior, Breastfeeding, Behavior, Fasting, Feeding Behavior, Motor Activity, Cigarette Smoking, Alcohol Consumption, Dietary Habits, Food Habits, Health Behaviors, Impulsivity, Breakfast, Smoking Cessation, Alcohol Drinking, Depressive Symptoms, Locomotor Activity |
| Anthropometry | Body Mass Index, Body Weight, Waist Circumference, Anthropometry, Weight, Waist-Hip Ratio, Skinfold Thickness, Body Size, Body Height |
| Nutrition Disorders | Morbid Obesity, Childhood obesity, Abdominal Obesity, Central Obesity, Prader-Willi Syndrome, Malnutrition, Visceral Obesity, Severe Obesity, Adolescent Obesity, Obesity Hypoventilation Syndrome, Pediatric Obesity, Vitamin D Deficiency, Starvation |
| Age Groups | Children, Adolescents, Elderly, Adults, Youth, Young Adults, Aged, Infant, Preschool Children, Low Birth Weight |
| Connective Tissue | Adipose Tissue, Visceral Adipose Tissue, Body Fat, Brown Adipose Tissue, Subcutaneous Adipose Tissue, White Adipose Tissue, Bone, Endothelium, Skeletal Muscle, Muscle |
| Metabolism | Energy Expenditure, Oxidative Stress, Metabolism, Lipolysis, Lipid Metabolism, Carbohydrate Metabolism, Energy Metabolism, Lipogenesis, Lipid Peroxidation, Renin-Angiotensin System |
| Demography | Prevalence, Mortality, Nutritional Status, Incidence, Morbidity, Ethnic Groups, Health Status, Community |
| Data Collection | Prevalence, Mortality, Incidence, Morbidity, Survey, Questionnaire, Questionnaires |
| Medicine | Epidemiology, Pediatrics, Surgery, Public Health, Rehabilitation, General Practice |
| Biochemical Phenomena | Body Composition, Lipolysis, Body Fat Distribution, Carbohydrate Metabolism, Lipogenesis, Lipid Peroxidation, Signal Transduction |
